# Ethical Challenges in Abortion Decision-Making: Perspectives from Perimenopausal Women’s Nursing Care

**DOI:** 10.17533/udea.iee.v43n1e01

**Published:** 2025-04-07

**Authors:** Tram Thi Bich Nguyen, Chi-Yin Kao, Yu-Yun Hsu, Riksa Wibawa Resna

**Affiliations:** 1 . M.Sc. Tutor/clinical Instructor. Email: nguyentbichtram17@duytan.edu.vn https://orcid.org/0000-0002-4358-4857 nguyentbichtram17@duytan.edu.vn; 2 . Ph.D, Associate Professor. Email: chiyinkao@mail.ncku.edu.tw. Corresponding author. https://orcid.org/0000-0002-5510-6668 chiyinkao@mail.ncku.edu.tw; 3 . Ph.D, Professor. Email: yuht12@mail.ncku.edu.tw https://orcid.org/0000-0001-6426-1021 yuht12@mail.ncku.edu.tw; 4 . Ph.D, Assistant Professor. Email: riksawibawa@stikesbanten.ac.id http://orcid.org/0000-0002-0596-3768 riksawibawa@stikesbanten.ac.id; 5 . Department of Nursing, College of Medicine, National Cheng Kung University, Tainan, Taiwan Department of Nursing College of Medicine, National Cheng Kung University Tainan Taiwan; 6 . Medical Simulation Center, Duy Tan University, Da Nang, Vietnam Medical Simulation Center Duy Tan University Da Nang Vietnam; 7 . Institute of Health Science Banten, Banten, Indonesia. Institute of Health Science Banten Banten Indonesia

Patient-centered care emphasizes patient autonomy and aligning treatment with their values. It integrates patients’ and families’ experiences into illness management, weighing risks and benefits. Ethical dilemmas arise when a pregnant woman’s interests conflict with the fetus’s potential life. The four-box approach offers a structured ethical framework, considering medical indications, patient preferences, quality of life, and contextual factors-each crucial to responsible, person-centered care.

Ethical Considerations in Women’s Abortion Decisions. Abortion is a complex issue with diverse ethical perspectives. Discussions require empathy, respect for differing views, and an understanding of women’s ethical challenges in making such decisions. The debate on abortion centers on a woman’s autonomy over her body versus the fetus’s right to life, raising ethical challenges in defining personhood and legal protection. Balancing the woman’s interests with the potential life of the fetus creates complex ethical dilemmas. Societal norms and regional legal differences shape abortion decisions, complicating ethical decision-making for women. A key concern is the impact on a woman’s physical and mental health, requiring informed consent, support, and counseling to address emotional and psychological effects.^1^

Ethical Challenges in Nursing Care for Perimenopausal and Unintended Pregnancies. Perimenopause, characterized by fluctuating hormones, still poses a risk of unintended pregnancy, typically starting in the early 40s but sometimes occurring earlier or later. An unintended pregnancy occurs when a woman does not intend to have more children or when the pregnancy occurs unexpectedly.^2^. Nearly 50% of such pregnancies end in abortion,^3^ leading to ethical dilemmas for patients and nursing practitioners. Nurses encounter complex ethical issues when caring for pregnant and peri-menopausal women, particularly those with unintended pregnancies. To navigate these challenges, nurses must understand ethical principles and their applications in healthcare. However, applying fundamental principles like autonomy, beneficence, non-maleficence, and justice can complicate ethical decision-making in these situations.

Integrating the Four-Box Approach into Patient-Centered Care for Unintended Pregnancies. Nurses face ethical dilemmas in unintended pregnancies, where the principle of autonomy allows mentally capable women to make their own treatment decisions. Person-centered care and shared decision-making emphasize integrating patient values. Providers must balance well-being (beneficence), harm prevention (nonmaleficence), and fair resource allocation (distributive justice). The four-box approach aids in making informed, morally justified treatment decisions by considering medical indications, patient preferences, quality of life, and contextual factors, ensuring responsible, person-centered care.

*Box 1: Medical Indications.* Pregnancy symptoms initially misattributed to menopause, such as weight gain, nausea, and breast tenderness, raise concerns about potential complications for perimenopausal women. Given their medical history and age, physicians are cautious about the heightened risks, including life-threatening emergencies. Abortion may provide emotional relief by alleviating stress over unexpected pregnancy and moral conflicts. Still, advanced maternal age also increases the risk of complications like ectopic pregnancy, fetal abnormalities, and gestational diabetes.^4^ However, the decision to abort can lead to ongoing emotional and moral struggles for women. It may strain relationships with partners or family members who wish to keep the baby, affecting family dynamics. Evaluating the clinical benefits and risks of abortion options is essential. These include surgical (uterine curettage aspiration) and medical (medications to induce contractions) methods.^5^ While surgical evacuation is traditional, medical abortion is a popular noninvasive alternative. A systematic review^5^ found pharmaceutical methods generally superior to surgical ones, highlighting the benefits of combination therapy over single interventions. Medical abortion using pills is a non-invasive, at-home option but carries risks such as heavy bleeding, cramping, and incomplete abortion ^6^. A study ^6^ of 26 076 women undergoing medical abortion found that 1.5% had ongoing pregnancies, 10.2% required surgical intervention, and 0.6% needed blood transfusions. Surgical abortion, including aspiration and dilation and curettage, is a quick outpatient procedure with a lower risk of incomplete abortion but increases the risks of infection and injury, especially with advanced gestational age. Factors like older age, a history of cesarean delivery, and inadequate dilation can further heighten the risk of complications.

*Box 2: Patient Preferences.* The unexpected pregnancy, initially confused with menopause, has created misunderstanding and stress for the women, evident in their emotional reaction to the positive test result. This heightened emotional state may impair cognitive processing. Their age and belief they were in menopause contributed to the initial confusion. The conflict between their moral beliefs against abortion and the recommended medical intervention further complicated their decision-making. A supportive decision-maker, such as a partner, family member, or friend, can significantly influence women’s decision-making by providing emotional support and valuable insights. Given differing views on the pregnancy, open communication and shared decision-making are essential.^7^ Involving a substitute decision-maker should be based on collaborative discussions that respect the woman’s autonomy and prioritize her preferences and values. This emphasizes the need for a comprehensive, patient-centered approach that addresses the situation’s medical, emotional, and moral aspects. In the face of unexpected pregnancy and initial confusion, women seek clarity and solutions that address their medical needs while maintaining family harmony and upholding strong moral values against abortion. For advanced-age women, health and family unity are vital priorities. Overall, they aim to make decisions that prioritize health, safety, family harmony, and respect for their beliefs. A patient-centered approach should incorporate these factors to support the decision-making process.

*Box 3: Quality of Life.* A multifaceted approach, focusing on health and overall well-being, is vital to sustaining or improving a woman’s quality of life. This includes regular check-ups for early detection and management of health issues, alongside emotional and mental support through counseling, support groups, and stress reduction strategies to help women navigate the emotional complexities of their situation.^8^ Shared decision-making is essential, involving women, their healthcare provider, and possibly their families to understand medical options thoroughly. Respecting women’s moral beliefs against abortion fosters trust and supports a patient-centered approach. Involving family, especially partners, can enhance support. Access to reproductive resources and future planning discussions, considering women’s age and health, are crucial. Collaboration with a multidisciplinary team ensures personalized care that aligns with women’s values and preferences.

*Box 4: Contextual Features.* Decisions on unintended pregnancy can strain family dynamics. Families often seek a resolution that prioritizes the woman’s health while considering the pregnancy. They may expect collaborative decision-making that balances medical advice with the desire to keep the baby. Depending on their agreement with the recommended course of action, women need emotional and practical support from their families, such as transportation, home assistance, and caregiving. Women’s moral beliefs against abortion often reflect cultural and religious values, making it essential for nurses to offer culturally competent care.^9^ Financial stability and access to specialized healthcare resources also play significant roles in decision-making. A holistic, patient-centered approach incorporating cultural competence, family dynamics, and financial and healthcare considerations is critical. Collaboration among healthcare providers, families, and support services helps women make decisions that align with their values and well-being.

Nursing Implications for Caring

Nurses must offer unbiased care, avoid personal beliefs, and follow professional standards. They should assess a woman’s decision-making capacity through clear communication, understanding, appreciation of personal implications, and reasoning. If abortion is refused, providers should consider it a temporary or permanent decision. Open discussions and empathetic support are essential, reassuring women that the procedure can be delayed to address concerns. Follow-up visits should explore options before deadlines, and if abortion is chosen, accurate information and legal access should be provided. Early termination by a qualified practitioner is safe, while delays increase risks. In conclusion, nurses should apply bioethical principles, using the four-box approach, to address the emotional and ethical challenges of caring for perimenopausal women with unintended pregnancies. This ensures patient-centered care by aligning medical indications, patient preferences, quality of life, and contextual features, helping to resolve value conflicts.
